# Palliative care in 9 children with neurodegeneration with brain iron accumulation

**DOI:** 10.1007/s10072-019-04099-5

**Published:** 2019-11-22

**Authors:** Tomasz Dangel, Tomasz Kmieć, Artur Januszaniec, Barbara Ważny

**Affiliations:** 1 Warsaw Hospice for Children Foundation, Agatowa 10, 03-680 Warsaw, Poland; 2 Department of Neurology and Epileptology, Children’s Memorial Health Institute, Warsaw, Poland

**Keywords:** NBIA, PKAN, Hospice, Ketogenic diet, Botulinum toxin, Quality care

## Abstract

**Aim:**

Evaluation of pediatric palliative home care of families with children suffering from neurodegeneration with brain iron accumulation (NBIA) and their parents.

**Material and methods:**

The children were treated at home by a multidisciplinary team. Densitometry was used to evaluate the condition of the skeletal system. Botulinum toxin was injected into the muscles in doses between 22 and 50 units/kg. The quality of palliative care was assessed on the basis of a specially designed questionnaire for parents.

**Results:**

The observations were performed on a group of 9 patients with NBIA. On admission, the median age of patients was 9 years (7–14). The average time of palliative home care was 1569 days (34 days–17 years). The median age at death (6 patients) was 11 years (7–15). The botulinum toxin injections gave the following results: reduction of spasticity and dystonia, reduction of spine and chest deformation, relief of pain and suffering, facilitation of rehabilitation and nursing, prevention of permanent contractures, and reduction of excessive salivation. Bone mineral density and bone strength index were reduced. Two patients experienced pathological fracture of the femur. The body mass index at admission varied between 9.8 and 14.9. In 7 cases, introduction of a ketogenic diet resulted in the increase of body mass and height. The ketogenic diet did not worsen the neurological symptoms. The parents positively evaluated the quality of care.

**Conclusion:**

Palliative home care is the optimal form of treatment for children with NBIA.

## Introduction

Neurodegeneration with brain iron accumulation (NBIA) is a heterogeneous group of rare diseases. There are two main types of NBIA: type 1, pantothenate kinase associated with neurodegeneration (PKAN), most often caused by the mutation of the PANK2 gene [1]; and type 2, mitochondrial protein–associated neurodegeneration (MPAN), caused by the mutation of the c19orf12 gene [2]. Besides those two types, there are other types of NBIA genes [3].

Pediatric NBIA occurs before the age of 10. There are two clinical presentations which depend on the age of the onset. The first one, which begins between the 1st and 2nd years of age, is characterized by muscle stiffness, generalized dystonia with torsional positioning of the feet and hands, and symptoms of oral-mandibular-vocal dystonia. In addition, there are parkinsonism symptoms and increased backward torso deflection, which impede breathing and nutrition, as well as anarthria and dysphagia. The second one, which begins after 5 years of age, has similar clinical presentation, but significantly slower progression.

The early-onset form is considered to be an incurable disease with a high risk of premature death and, as such, is an indication for pediatric palliative care [4].

In Poland, the first NBIA patient was admitted to the Warsaw Hospice for Children (WHC) in 1998.

## Objective

The aim of the study is to assess pediatric palliative home care as a new approach for children with NBIA and their families.

## Material and methods

The clinical data was obtained from the WHC database. The ketogenic diets were designed with Diet-5d program of the National Food and Nutrition Institute. Densitometry was performed at the Department of Biochemistry and Experimental Medicine at the Children’s Memorial Health Institute in Warsaw, Poland. The total body (without the head) (TB BMD) bone mineral density and the bone mechanical strength index (TB BMC/LBM) were examined. To treat spasticity, botulinum toxin (Dysport, dosage between 22 and 50 units/kg) was injected into the muscles (by the third author). There was a two-stage effectiveness evaluation protocol—2 weeks and 3 months after the procedure, which was carried out by the WHC physiotherapist.

Deep brain stimulation was conducted by the Department of Neurosurgery, the Institute of Psychiatry and Neurology in Warsaw, Poland.

The quality of palliative care was assessed on the basis of a feedback questionnaire for parents of patients who died at home under the WHC care [5]. For the families of the living children, a new version of the quality questionnaire was designed (not yet published). The study covers the period between 1998 and 2018.

## Results

There were nine patients with early-childhood NBIA. NBIA type 1 (PKAN) was diagnosed in 8 (codes A, B, C, D, E, F, G, I), and NBIA type 2 (MPAN) in one (code H). All of the patients were referred to the WHC from the Department of Neurology and Epileptology at the Children’s Memorial Health Institute in Warsaw, Poland. The referral was given by the second author of the present article.

The age of the patients on admission to the WHC was 9 years in average (from 7 to 14 years) (Table 4). It should be noticed that the disease occurred a couple of years earlier and its evolution was very fast, depending on the type of the gene mutation. The average period of home palliative care lasted 1569 days (from 34 days to 17 years), i.e., over 4 years. Six children passed away, and three are still alive. The average age of death was 11 years (from 7 to 15 years).

In all the cases, the treatment was carried at the patients’ homes. Five of the patients died at home, and one was admitted to the hospital, due to parental decision, where he passed away after 6 days (A). One child (G) was discharged from the WHC as the result of parental request.

The list of the symptoms present in the 9 children with NBIA treated in the home hospice is presented in Table 1.

**Table 1:** Symptoms in 9 children with NBIA provided with palliative care

No	Symptom/pathology	Frequency (%)	Treatment
1.	Spasticity	100	Pharmacotherapy, botulinum toxin, deep brain stimulation
2.	Dystonia	100	
3.	Dysphagia	100	Intragastric feeding, ketogenic diet
4.	Cachexia from malnutrition	100	
5.	Anarthria	100	Nonverbal forms of communication
6.	Mental retardation	100	
7.	Restlessness, agitation	100	Midazolam, phenobarbital, clonazepam*
8.	Constipation	100	Enema, laxatives, fiber
9.	Inflammation around fistulas (gastrostomy, tracheostomy)	100	Ointments, laser therapy, surgical treatment
10.	Pain	100	Analgesics (paracetamol, tramadol, methadone)
11.	Spine and chest deformation	100	Botulinum toxin, rehabilitation
12.	Fever	89	Paracetamol, metamizol, physical cooling
13.	Gastric retention	89	Metoclopramide, trimebutine, diet modification
14.	Vomiting	78	Pharmacotherapy
15.	Sleep disorders	78	Melatonin
16.	Upper respiratory tract infections	78	Inhalations, inosine pranobex
17.	Excessive sweating	67	
18.	Abdominal distension	67	Drotaverine hydrochloride, pancreatin, simeticone
19.	Bedsores	67	Anti-bedsore mattress, bandages
20.	Fungal infections (oral cavity, skin)	67	Pharmacotherapy, ketogenic diet, probiotics
21.	Hypoxia (SaO_2_ < 90%)	56	Oxygen therapy
22.	Micturition disorders	56	Bladder catheterization, pharmacotherapy
23.	Lower respiratory tract infections	56	Antibiotics, physiotherapy (cough assistor)
24.	Digestive tract infections	56	Pharmacotherapy, rehydration, electrolytes by the enteral route
25.	Limb arthritis (swelling)	56	Pharmacotherapy
26.	Food intolerance	44	Diet modification, pharmacotherapy, probiotics
27.	Excessive salivation	33	Botulinum toxin (into salivary glands)
29.	Subcutaneous edema	33	Furosemide
30.	Atelectasis	33	Physiotherapy (cough assistor), oxygen
31.	Urinary tract infections	33	Antibiotics, furazidin, cranberry
32.	Ear infections	33	Antibiotics, drains, analgesics
33.	Laryngeal dystonia and stridor	33	Tracheostomy
34.	Eye inflammations	22	Antibiotics (locally)
35.	Gingival hyperplasia	22	
36.	Bone fractures	22	Orthopedic treatment
37.	Gastroesophageal reflux	22	Omeprazole, dexlansoprazole, metoclopramide (surgical treatment was not possible because of a collision with stimulators)
38.	Subcutaneous abscesses	11	Ichthyol ointment, drain

*According to our practice, these drugs are the most effective ones

Pharmacotherapy, supervised by the second author, included baclofen, diazepam, estazolam, phenobarbital, gabapentin, hemineurin, carbidopa, clonazepam, clonidine, valproic acid, levodopa, midazolam, promazin, tetrazepam, and tizanidine. The patients required 8 (3–10) of those medications in average [6].

**Fig. 1:**
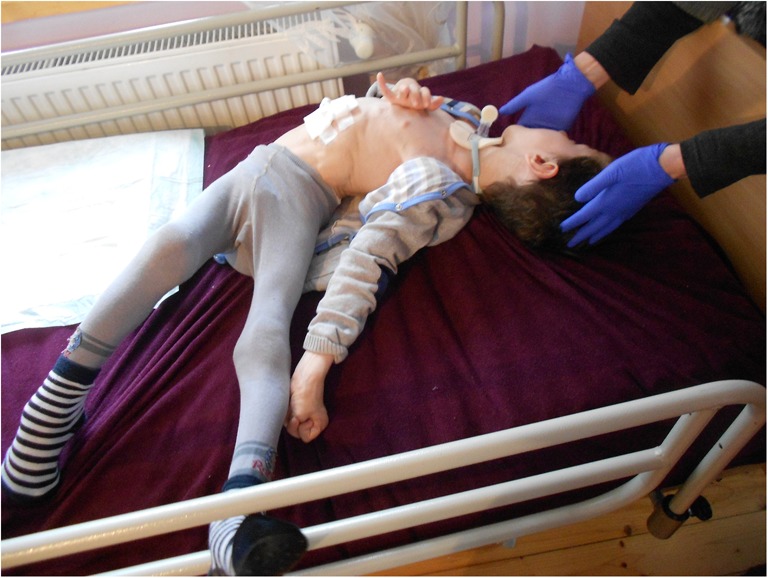
Patient A at admission to WHC (BMI 12.3; percentile 0)

**Fig. 2:**
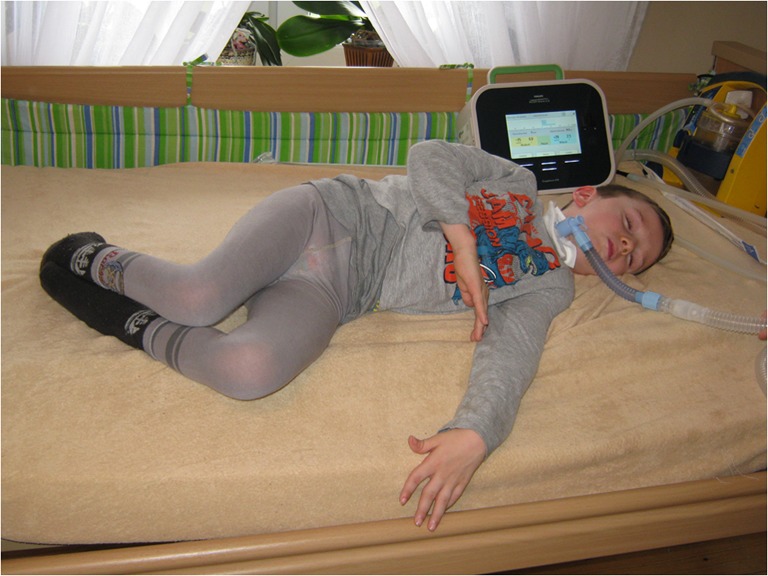
Patient A 1 year and 3 months since admission to WHC (BMI 14.3; percentile 11, weight and height gain + 4.1 kg and + 7 cm, respectively). The effect of treatment with botulinum toxin and a balanced ketogenic diet. Visible cough assistor

The result of the botulinum toxin treatment is summarized in Table 2. The first substantial improvement of reduction of muscle tension and salivary secretion appeared after 7 days and reached its greatest efficacy at 14–60 days. Muscle relaxation lasted from 3 to 18 months. The beneficial effects covered the following: reduction of spasticity, dystonia, salivation, spinal and thorax deformity, pain and suffering, and prevention of permanent contractures, as well as facilitation of rehabilitation and daily care Figs 1 and 2.

**Table 2:** Botulinum toxin treatment results in 8 patients with NBIA*

No	Code	Number of treatments		Average effectiveness (%)	Duration of effect (months)
		Effective	Ineffective		
1.	A	10	0	80	3–4
2.	B	7	0	60	5
3.	C	6	0	90	8
4.	D	2	0	70	18
5.	E**	2	0	60	
6.	F	11	0	70	6
7.	G	9	1	70	6
8.	H	2	2	50	9

*The first patient (I) treated between 1998 and 2000 is not included because the treatment with botulinum toxin was initiated at the WHC in 2002

**The patient (E) died after a month

Deep brain stimulation was introduced in 2 patients (B, F). In both cases, a satisfying reduction of dystonia and spasticity was obtained. The treatment lasted 3 years (B) and 8 years (F).

Laryngeal dystonia and stridor occurred in 3 patients. Tracheostomy was performed in two of them, which prolonged their life by 2 (A) and 3 years (D). The third child (C), also scheduled for the procedure, died before hospital admission.

Six patients underwent dental treatment under general anesthesia at the WHC Dental Clinic.

In bacteriological studies of respiratory and digestive tracts, the following pathogens have been detected: *Acinetobacter* spp., *Citrobacter koseri*, *Delftia acidovorans*, *Enterobacter cloacae*, *Enterobacter* species, *Escherichia coli*, *Haemophilus influenzae*, *Klebsiella oxytoca*, *Klebsiella pneumoniae*, *Moraxella catarrhalis*, *Neisseria* species, *Pseudomonas aeruginosa*, *Serratia marcescens*, *Staphylococcus aureus*, *Staphylococcus haemolytic*, *Stenotrophomonas maltophilia*, *Streptococcus agalactiae*, *Streptococcus pneumoniae*, *Streptococcus pyogenes*, *Streptococcus viridans*. Moreover, in mycological studies, the growth of *Candida albicans*, *Candida famata*, *Candida glabrata*, *Candida kefyr*, and *Candida parapsilosis* was observed.

Densitometry and the index of bone mechanical strength were performed in 4 patients (Table 3). Pathological fractures of the femur occurred in 2 patients and required orthopedic intervention. On admission, the dose of vitamin D_3_ was increased to achieve the serum 25(OH) D level of 50–80 ng/ml.

**Table 3:** Densitometry results in children with NBIA during palliative care

No.	Code	Mineral bone density		Index of bone mechanical strength		Pathological fractures
		1st measurement	2nd measurement	1st measurement	2nd measurement	
1.	C	− 3.2	− 3.4	− 4.2	− 2.9	No
2.	D	− 3.3	− 3.4	− 2.9	− 2.7	No
3.	G	− 3.8	− 1.7	− 1.1	− 1.5	Yes
4.	H	− 3.2	− 3.2	− 3.2	− 3.1	Yes

**Fig. 3:**
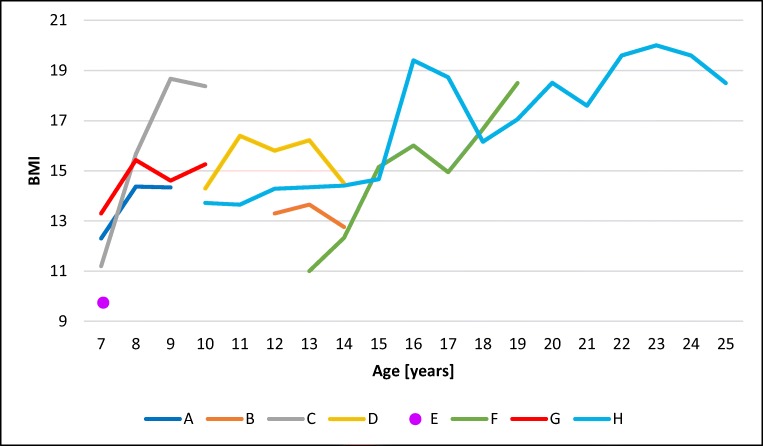
BMI values in consecutive years of life in 8 patients with NBIA

Table 4 presents the values of anthropometric measurements on admission to the WHC. Eight out of 9 patients were severely cachectic. Figure 3 presents the values of the body mass index (BMI) of the 8 cachectic patients.

**Table 4:** Anthropometric measurements on admission to the WHC

No	Code	Age (years)	Mass (kg)	Growth (cm)	BMI*	Percentile BMI
1.	A	7 ^8^/_12_	16.5	114	12.3	0
2.	B	12 ^1^/_12_	25.2	137	13.3	0
3.	C	7 ^7^/_12_	14.5	112	11.2	0
4.	D	10 ^6^/_12_	28.5	140	14.3	5
5.	E	6 ^11^/_12_	15.4	124	9.8	0
6.	F	13 ^10^/_12_	24.3	148	11	0
7.	G	7 ^2^/_12_	13.3	99	13.3	2
8.	H	8 ^1^/_12_	22	130	13	1
9.	I	8 ^10^/_12_	18	110	14.9	22

*Body mass index

Dysphagia as the fundamental obstacle in the adequate nutrition regime was treated by the gastric fistula establishment (8 patients). In one case (I), despite symptoms of dysphagia, the parents did not agree on gastrostomy insertion. The orally fed child died due to aspiration.

Another crucial aspect of severe malnourishment was lack of a properly balanced diet. Before admission, the patients received standard pre-prepared industrial or liquidized “table food.” On admission, the parents were offered two options: an industrial, high-carbohydrate diet or a balanced ketogenic diet individually designed by the first author (Table 5). The ketogenic diet based on natural products and supplements was chosen by 8 families, and well tolerated by 6 patients (A, C, E, F, G, H). In 2 cases (A, D), small adjustments were necessary in the last stage of life. Seven children obtained significant weight and height gain (except E). No negative effect of ketogenic diet on neurological symptoms was observed.

**Table 5:** Balanced ketogenic diet characteristics in 8 NBIA patients*

No.	Code	Protein (g/kg)	Energy (kcal/kg)	Energy balance (%)			Ketonuria	Fasting glucose (mg/dl)
				Fat	Carbohydrates	Protein		
1.	A	1.9	80	82	8	10	+/−	78
2.	B	2.3	61	79	6	15	+/−	85
3.	C	2.4	86	84	5	11	+	88
4.	D	1.9	53	83	3	14	+++	88
5.	E	1.8	89	91	1	8	+/−	75
6.	F	2.4	62	82	3	15	+	72
7.	G	3.2	105	81	7	12	+++	76
8.	H	1.7	51	86	1	13	+++	81

*The first patient (I) treated between 1998 and 2000 is not included because the treatment with ketogenic diet was initiated at the WHC in 2004

The content of pantothenic acid in the ketogenic diet ranged from 6 to 14.5 mg/day, which accounted for 150–290% of the daily intake recommended by the National Food and Nutrition Institute.

Primary and secondary causes of death in 6 deceased patients are presented in Table 6. Neither terminal/palliative sedation nor intravenous opioid analgesics were required.

**Table 6:** Primary and secondary causes of death

No	Code	Age at death (years)	Cause of death	
			Primary	Secondary
1.	A	9 ^8^/_12_	Atelectasis	Spine deformation
2.	B	14 ^8^/_12_	Atelectasis	Spine deformation
3.	C	10 ^10^/_12_	Airway obstruction	Laryngeal dystonia
4.	D	14	Apnea	?
5.	E	7	Cachexia from malnutrition	Dysphagia
6.	I	10 ^4^/_12_	Aspiration	Dysphagia

Nine questionnaires filled in by the parents of 5 deceased children were obtained (mothers, 5; fathers, 4). In addition, four questionnaires were obtained from the parents of 2 living patients.

Parental needs were identified by asking about their expectations related to hospice care. The respondents mentioned: we would receive medical assistance (*n* = 12), my child would not suffer anymore (*n* = 12), we would receive psychological support (*n* = 9), our helplessness as caretakers would decrease (*n* = 8), we would receive spiritual support (*n* = 8), my child would feel safe (*n* = 7), we would receive financial support (*n* = 5), the hospice would help make formal arrangements after my child’s death (*n* = 1). During hospice care, all families required Foundation’s WHC financial support.

The frequency of home visits and quality of the childcare guidance provided by WHC physicians and nurses were assessed as adequate by all parents (*n* = 13).

The biggest parental worries and struggles identified during palliative home care were as follows: helplessness (*n* = 11), controlling pain and other symptoms (*n* = 10), my own emotional exhaustion (*n* = 6), fear (*n* = 5). Less frequently mentioned issues are as follows: feeling of imprisonment at home (*n* = 2), my own physical exhaustion (*n* = 2), making decisions (*n* = 2), my own inability to provide care (*n* = 1), spiritual crisis (*n* = 1).

In parents’ opinion, WHC was able to help with the following: pain and other symptoms control (*n* = 10), countering helplessness (*n* = 6), emotional exhaustion (*n* = 4), fear (*n* = 2), making decisions (*n* = 2), spiritual crisis (*n* = 1), and their own inability to provide care (*n* = 1).

However, there were problems which could not be solved such as the following: fear (*n* = 3), helplessness (*n* = 3), physical exhaustion (*n* = 2), emotional exhaustion (*n* = 2), and feeling of imprisonment at home (*n* = 1).

Two respondents stated that some medical decisions were difficult for them to accept. In the first case (A), parents were concerned about the introduction of new methods of treatment: diet, cough assistor, some medications, and the replacement of the tracheostomy tube at home. In the second case (B), parents questioned the decision of withdrawing from life-prolonging treatment in the terminal phase.

Only three parents felt prepared for the death of their child; all of them confirmed that the role of the hospice was helpful.

The parents evaluated the effectiveness of symptom control as follows: nine of them (representing 5 patients) felt that the child had suffered rarely, but occasionally symptoms had increased although this was relieved with appropriate treatment; and four parents (representing patients A, I) stated that the child had suffered most of the time, though occasionally the treatment had resulted in relief.

The numeric scale was used to evaluate parental satisfaction with the hospice care (from 0, “I am very dissatisfied,” to 10, “I am very satisfied”). The average rating was 10.

Using the same scale, the parents rated the quality of care provided by physicians (9.9), nurses (9.9), social workers (9.8), chaplains (9.8), psychologists (10), physiotherapists (9.7), and volunteers (9.8).

Two parents (from the same family) and four siblings (from four different families) took part in bereavement support programs. Four children (from four families) attended the support program for siblings of living patients.

## Discussion

A group of experts associated with the TIRCON research project (Treat Iron-Related Childhood-Onset Neurodegeneration) recommends early involvement of palliative care team after the diagnosis of the type 1 PKAN [7]. However, in the available literature, there are no reports regarding the use of palliative care in this group of patients.

It should be stressed that NBIA is significantly different from other neurodegenerative disorders of childhood. The NBIA patients are permanently aware of their symptoms and suffering, although they cannot verbally communicate pain which is secondary to dystonia (Table 1). Therefore, based on the authors’ experience, palliative care embracing all treatments described in the present article is the only effective approach. What is also worth emphasizing is the fact that these treatments can be successfully provided at home where the children feel safer and more comfortable than at hospital.

The symptoms which cause suffering in children with NBIA are secondary to neurodegeneration. Dystonia as the dominant symptom leads to deformation. Different types of pain are caused by spasticity (muscular and joint pain), fractures (bone pain), headaches (vegetative pain), micturition disorders and constipation (visceral pain), esophagitis due to gastroesophageal reflux, and other inflammatory conditions (inflammatory pain). Diagnosing pain as a cause of restlessness is difficult because of dystonia, anarthria, and intellectual impairment. In such cases, the first-line treatment included pain relief medications. Further management depended on the patient’s response.

Almost all NBIA children were referred to the WHC in a state of extreme malnutrition and cachexia. The child with the lowest BMI (9.8) died after 34 days. In the remaining patients with higher BMI values (11–14.3), a positive response to nutritional treatment was observed.

The TIRCON expert group recommendation against a ketogenic diet for all patients with PKAN [7] was based on the only published study which was conducted on mice [8]. Their results show that pantothenic acid prevents the negative effects of the ketogenic diet in PKAN mice. For that reason, the ketogenic diet used in 8 WHC patients contained large amounts of pantothenic acid (Table 7).

**Table 7:** Pantothenic acid recommended dietary allowances (RDA) and content in the diet of 8 patients with NBIA

No.	Code	Pantothenic acid (mg)	
		RDA	Content in diet
1.	A	4	10.7
2.	B	5	14.5
3.	C	4	7.5
4.	D	5	12.3
5.	E	4	6
6.	F	5	8.8
7.	G	4	7.8
8.	H	5	8.2

Conversely, there was one case report published which described the positive impact of the ketogenic diet in a patient with NBIA [9]. Our experiences are similar and prove the positive effects of this type of nutritional treatment. In our previous study, we demonstrated that the ketogenic (low-carbohydrate) diet in WHC patients is more beneficial than the industrial, high-carbohydrate diet because it reduces the risk of candidiasis and intestinal permeability [10].

Respiratory rehabilitation is the second most important form of therapy necessary to avoid premature death of the patient. Its effectiveness increases with the use of the botulinum toxin (paraspinal muscle relaxation) and a cough assistor with the function of lung expansion (prevention and treatment of atelectasis of the lungs).

Symptomatic treatment in palliative care can be effectively carried out at home by a hospice team of adequately trained doctors, nurses, and physiotherapists. It is necessary to maintain close cooperation with a neurologist consultant and a center that conducts deep brain stimulation.

Home palliative care can be effectively carried out if parents or carers accept and respect the ethical principles, standards, and treatment procedures, and follow medical advice. Hospital admission of a child in a terminal phase is associated with the risk of harmful life-prolonging therapy. The end of life plan and do-not-resuscitate protocol should be established before the referral to a palliative care program [11].

Parents or carers who provide home-based palliative care often experience chronic stress and a range of mental, emotional, spiritual, physical, and financial problems. Hospice workers (i.e., psychologist, chaplain, social worker) should be able to recognize such problems and provide appropriate support. The parents of our patients rated highly the quality of home palliative care, which confirms its effectiveness.

## Conclusions

1. The situation of children with early forms of PKAN and MPAN is particularly difficult because they cannot verbally communicate their suffering, despite being conscious and aware.
2. Home palliative care is the most effective form of treatment. Symptom control is possible at home and does not require intravenous therapy.
3. In cases of parental action contrary to the child’s best interest, the hospice physician is obliged to notify the family court. The example of such situation is inappropriate nutrition leading to starvation, or oral feeding which may cause aspiration and death.
4. Children with BMI ≥ 11 may survive in response to nutritional therapy. Death from starvation occurs below this value. Lifespan depends on the response to nutritional treatment.
5. In the case of gastroesophageal reflux, antireflux surgery should be performed prior to the implantation of brain stimulators.
